# Neural network architectures and normalization techniques for automated sleep stage classification using rodent EEG and EMG signals

**DOI:** 10.1371/journal.pone.0346294

**Published:** 2026-04-23

**Authors:** Jinyoung Choi, Hankil Oh, Minkyu Ahn

**Affiliations:** 1 Department of Anesthesiology, Mass General Brigham, Department of Anaesthesia, Harvard Medical School, Boston, Massachusetts, United States of America; 2 Department of Computer Science and Electrical Engineering, Handong Global University, Pohang, Republic of Korea; Suez Canal University Faculty of Medicine, EGYPT

## Abstract

Accurate sleep stage classification in animal models is crucial for translational sleep research, enabling the study of mechanistic pathways and therapeutic interventions. Because manual scoring is labor-intensive and variable, artificial neural networks are increasingly used for automation. However, few models are tailored for animal sleep staging, and direct cross-model comparisons under consistent conditions remain limited. We presents a systematic evaluation of three representative neural architectures for automated sleep stage classification using rodent electroencephalogram and electromyogram: a conventional 1-dimensional convolutional neural network (1D-CNN), a 2-dimensional convolutional neural network (AccuSleep), and a convolutional neural network combined with bidirectional long short-term memory (DeepSleepNet). Performance was assessed under within-subject and cross-subject validation frameworks, comparing raw input, z-scoring, and mixture z-scoring. Both 1D-CNN and DeepSleepNet consistently outperformed AccuSleep, particularly for Rapid Eye Movement (REM), where AccuSleep exhibited marked deficits plausibly attributable to class imbalance. Class-wise analysis confirmed stable Non-Rapid Eye Movement (NREM) classification across models, while AccuSleep showed reduced robustness in REM and Wake. Normalization effects were model-dependent: raw data yielded superior outcomes for 1D-CNN and DeepSleepNet, whereas AccuSleep showed modest improvement in Wake detection under mixture z-scoring. Comparison with human electroencephalogram literature indicated that DeepSleepNet’s advantage over 1D-CNN is more pronounced in human datasets (especially NREM 1), likely reflecting differences in sleep architecture. These findings highlight the suitability of simpler CNNs for rodent sleep stage classification and underscore the importance of aligning preprocessing strategies with model architecture and data characteristics.

## Introduction

Sleep is a fundamental biological process essential for cognitive, physical, and emotional health [1,2]. Insufficient or disrupted sleep is associated with impairments across cognition and immune function, metabolic dysregulation, and increased risk for neuropsychiatric and neurodegenerative conditions [1,3–6]. As these links grow increasingly clear, understanding the physiology sleep has become a central priority in biomedical research [6].

Sleep stage classification provides an objective framework for interrogating sleep architecture. In humans, sleep is categorized into non-rapid eye movement (NREM; stages N1-N3) and rapid eye movement (REM) based on characteristic patterns across electroencephalogram (EEG), electromyogram (EMG), and electrooculogram (EOG) [7]. Rodent, however, exhibit polyphasic, fragmented sleep with short, frequent episodes, differing markedly from consolidated human sleep [8]. Animal models offer complementary leverage for mechanistic studies and for evaluating the consequences of sleep disruption under controlled experimental conditions [9–11].

Historically, experts have manually scored polysomnography (PSG) data To assign stages [7]. Although manual scoring \ remains the clinical standard, it is time-consuming, labor-intensive, and subject to inter-rater variability [12]. To address these limitations, automated methods leveraging machine learning and deep neural networks have become a focal point. Convolutional neural networks (CNNs) are particularly effective at extracting discriminative features from EEG/EMG [13–15], and their combination with recurrent units (e.g., Recurrent neural networks, RNN and Long short-term memory, LSTM networks) enables models to capture temporal dependencies critical for staging [16,17]. More recently, attention-based and transformer-based architectures have been explored to further enhance representational capacity [18–20].

Despite these advances, applying neural networks to rodent data remains challenging. The species-specific sleep architecture—short and frequent episodes without long consolidated bouts—limits the direct transfer of models optimized for human, five‑class staging to rodent, three‑class settings [8,15]. Moreover, the number of rodent‑specific architectures is small: for instance, one attention‑based model has been proposed [21] and one transformer‑based approach has been reported [20], while most animal studies still rely on conventional CNNs or CNN–(bi)LSTM hybrids [17,22–27]. A second barrier is the lack of standardized, large‑scale rodent datasets, which hampers reproducibility and prevents like‑for‑like comparisons; many studies cite results from different papers rather than reconstructing models under identical conditions [17,21,24,25,28–30]. A third unresolved issue concerns normalization: approaches such as mixture z‑scoring have been proposed to mitigate subject variability and class imbalance [31], but their general utility across architectures remains unclear because they are often evaluated only within the specific studies that introduced them [31]. Given these gaps, our study pursues a focused, reproducible comparison of three representative architectures that collectively span the dominant design space in rodent sleep staging:

1. 1D-CNN—a widely employed architecture for time-series analysis due to their ability to capture local temporal dependencies and hierarchical feature representations [32];
2. 2D-CNN (AccuSleep)—a spectrogram‑based approach that operationalizes mixture z‑scoring to address distributional shift and subject variability [31];
3. CNN + biLSTM (DeepSleepNet)—a hybrid architecture designed to capture temporal context via bidirectional LSTM, adapted here to dual‑channel EEG + EMG [33].

These three models were selected not merely as examples, but as canonical representatives of how rodent sleep staging is currently performed in practice: (i) raw vs. spectrogram inputs, (ii) convolution‑only vs. convolution+temporal modeling, and (iii) with vs. without model‑specific normalization. By reconstructing AccuSleep, DeepSleepNet, and a representative multi-layer 1D-CNN using open‑source code, we provide direct, controlled comparisons under the same dataset [31] and identical validation schemes. This design enables us to answer three questions of practical and scientific significance:

- Architecture efficacy: In polyphasic rodent sleep, do simpler CNNs suffice, or does temporal modeling (biLSTM) confer measurable benefits relative to spectrogram‑based 2D‑CNN?
- Normalization utility: Does mixture z‑scoring or conventional z-scoring improve performance across architectures and stages, or can raw inputs be preferable for CNN/CNN + biLSTM?
- Generalization context: How do these findings compare with results from other datasets (e.g., Sleep‑EDF database), and how might dataset scale/class imbalance modulate outcomes [31–33].

To ensure rigor and reproducibility, we evaluate performance under two complementary frameworks: within-subject validation (per-animal training/testing) to probe stability within individual, and cross-subject validation (train on one, validate across others) to assess generalization across animals. Together, these analyses allow us to disentangle model‑specific characteristics from preprocessing effects, quantify their relative contributions to classification accuracy and F1, and articulate evidence‑based guidance for selecting architectures and normalization strategies in rodent sleep research [15,17,28,30,31]. Ultimately, our goal is to recenter the field on transparent, reproducible comparisons that align architecture and preprocessing with the species‑specific sleep structure, class distribution, and dataset scale most relevant to animal studies.

## Methods

### Dataset

**Fig 1:**
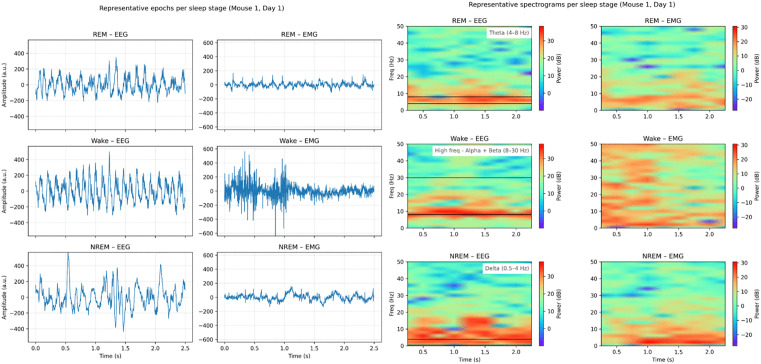
Representative raw EEG and EMG epochs with corresponding spectrograms for each sleep stage. The first two columns display time-series plots of raw EEG and EMG signals for each sleep stage, respectively. The last two columns show spectrograms derived from the same EEG and EMG data, with horizontal black lines indicating stage-specific dominant frequency bands: REM exhibits theta‑band prominence, Wake shows strong EMG activity across higher frequencies, and NREM is characterized by low‑frequency dominance.

We used the dataset from AccuSleep study **https://doi.org/10.17605/OSF.IO/PY5EB** [31,34], comprising sleep EEG and EMG recordings from 10 mice, each with five sessions. Each sessions contains 4 hours of data collected between 1 PM and 5 PM after a 2-hour habituation period. Signals were sampled at 512 Hz and annotated every 2.5 seconds into Wake, NREM, and REM. Representative raw EEG/EMG epochs with spectrograms are shown in Fig 1.

On average, the dataset’s stage distribution was approximately 55% NREM, 35% Wake, and 10% REM. To address class imbalance in training, we oversampled minority classes using synthetic minority oversampling technique (SMOTE) [35]. until they matched the number of NREM epochs per recording. We inspected the top 0.5% of epochs by maximum amplitude to identify noise; most artifacts reflected minor EEG contamination by EMG (Supplementary S1 Fig). Because such artifacts are common and potentially useful for generalization, no epochs were removed.

### Neural network architecture

**Fig 2:**
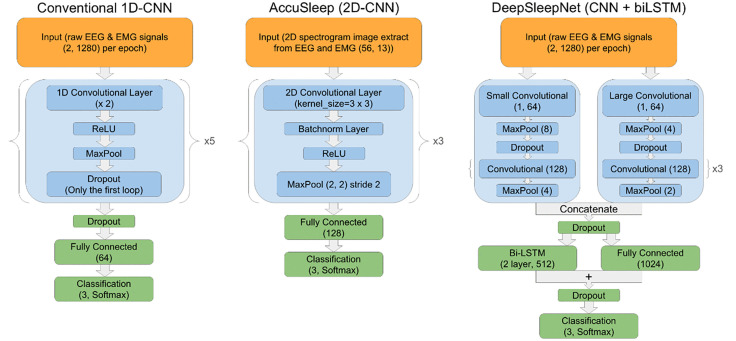
Neural network architectures for sleep stage classification.

In this study, we employed three distinct neural network architectures (Fig 2) for sleep stage classification, each reflecting a conventional strategy for feature extraction and classification using EEG and EMG signals.

### 1D-CNN

Inputs are raw EEG and EMG per epoch with shape ([2], 1280). The network has five blocks, each comprising two 1D convolutional, Rectified Linear Unit (ReLU), and max pooling. Filter counts and kernel sizes increase across the blocks to progressively capture features; dropout is applied after the first block only. Convolutional outputs feed a 64-unit fully connected (FC) layer, followed by a Softmax classifier for three stages. CNN is known to capture temporal patterns in the data, enabling effective feature extraction in time-series data. This baseline architecture is widely used in sleep staging [32,36,37], and often embedded in hybrid models [33,38,39].

### 2D-CNN (AccuSleep)

We computed EEG spectrograms using 0–20 Hz fully and 20–50 Hz even-indexed frequency bins, emphasizing sleep-relevant low frequencies. EMG was band-pass filtered 20–50 Hz and its RMS appended as a constant vector at the end of EEG frequency axis to form a 2D image. The 2D-CNN comprises three convolutional blocks, each with Conv2D, BatchNorm, ReLU, and max pooling. Feature maps increase across blocks; outputs feed FC‑128 and Softmax‑3. AccuSleep is notable for including mixture z‑scoring to address distributional shift and subject variability [31].

### CNN + biLSTM (DeepSleepNet)

We adapted DeepSleepNet [33]—originally single-channel EEG—to incorporate raw EEG + EMG dual‑channel inputs. The CNN has two branches: a “small” branch to detect temporal patterns (larger pooling: e.g., 8 and 4) and a “large” branch to extract frequency components (smaller pooling: e.g., 4 and 2). Each branch has four convolutional layers. Branch outputs are concatenated and passed through a biLSTM (two layers, 512 units), then FC-1024, element-wise addition, dropout and SoftMax-3.

### Normalization methods

We compared three input conditions: (i) raw (no normalization), (ii) standard z-scoring, and (iii) mixture z-scoring. Standard z-scoring normalizes features using the global mean and variance. Mixture z-scoring standardizes features of the EEG and EMG while accounting for class imbalance by using a subset of labeled data from each subject. It requires prior information on the proportion (*w*) of epochs as well as the mean (*μ*) and variance (*σ*) of feature values for each class, which are derived from the training data. This information is used to normalize the input features (*x*) according to the equation (1), thereby mitigating class imbalance and subject variability.


$$Z=\frac{x-w^{T}\mu }{\sqrt{w^{T}\left(\sigma^{\text{2}}+s\right)}},\;s={\left(\mu -w^{T}\mu \right)}^{\text{2}}$$ (1)

We applied these methods to all three models to enable fair, like-for-like comparisons under identical preprocessing.

### Training and hyperparameters

The training was conducted over 50 epochs for the AccuSleep and 25 epochs for the 1D-CNN and DeepSleepNet models, with a consistent batch size of 128. Learning rates were set at 0.0001 for the 1D-CNN, 0.015 for AccuSleep (with a 15% reduction per epoch), and 0.05 for DeepSleepNet. AccuSleep was optimized using Stochastic gradient descent (SGD) with 0.9 momentum, whereas the Adam optimizer was applied to the 1D-CNN and DeepSleepNet models.

### Validation methods

#### Within subject validation.

For each mouse, three recordings were used for training, one for estimating normalization parameters (class-wise mean/variances for mixture z-scoring, overall mean/variance for z-scoring), and one for validation. Under the raw condition, the normalization recording was excluded from training to ensure parity across conditions.

#### Cross subject validation.

All five recordings from one mouse were used for training. For each of the remaining nine mice, four recordings were used for validation, and one recording was reserved solely to estimate normalization parameters (mixture or z-scoring), and not included in validation for the raw condition.

### Statistical analysis

Accuracy and F1-score were the primary metrics. In within-subject validation, we obtained 10 samples per metric per condition. In cross-subject validation, 90 samples per metric. We used one-way analysis of variance (ANOVA) to assess differences among the models, and Tukey’s honestly significant difference (HSD) for post-hoc pairwise comparisons when appropriate.

## Results

### Overall model performance across normalization methods

#### Within-subject validation.

**Fig 3:**
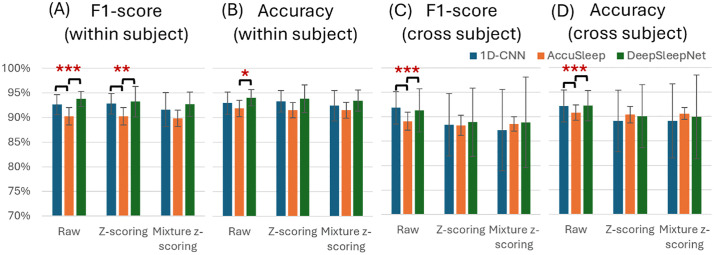
Overall performance comparison of neural network models for sleep stage classification. One-way ANOVA was conducted to compare the performance of the three networks under the same condition (comparisons limited to adjacent bars; *p < 0.05, **p < 0.01, ***p < 0.001). Each bar represents the mean performance with standard deviation.

DeepSleepNet showed the highest average accuracy (94.1%) and F1-score (93.8%) when trained on raw data (Table 1, last two columns). However, statistical comparisons indicated no significant differences between DeepSleepNet and 1D-CNN (Fig 3A, 3B). Both models significantly outperformed AccuSleep in F1-score when using raw or z-scored data (F(2, 27) = 6.67, p = 0.004, one-way ANOVA, Fig 3A). AccuSleep trained on raw data also demonstrated significantly lower accuracy compared to DeepSleepNet (p = 0.0357, post-hoc pairwise comparison, Fig 3B).

**Table 1:** Performance Comparison of 1D-CNN, AccuSleep, and DeepSleepNet Using Raw, Z-Scored, and Mixture Z-Scored Data for Within-Subject and Cross-Subject Validation.

Within-subject		REM			Wake			NREM			Accuracy	F1-score
		Precision	Recall	F1-score	Precision	Recall	F1-score	Precision	Recall	F1-score		
1D-CNN	None	91.7	95.6	**93.6**	87.4	93.9	90.3	96.7	91.7	94.0	92.9	92.6
	Z-scoring	94.0	92.6	93.2	89.4	92.5	**90.8**	95.3	93.6	**94.4**	93.3	92.8
	Mixture z-scoring	87.7	95.3	90.9	89.4	91.9	90.4	95.6	91.9	93.5	92.4	91.6
AccuSleep	None	88.4	87.8	**88.0**	89.2	89.4	**89.2**	93.7	93.6	**93.6**	91.9	90.3
	Z-scoring	87.5	86.4	86.9	89.0	89.5	89.2	93.4	93.3	93.3	91.5	89.8
	Mixture z-scoring	85.8	89.0	87.3	88.7	89.3	89.0	93.8	92.7	93.3	91.5	89.9
DeepSleepNet	None	95.3	94.6	**94.8**	91.7	91.9	**91.6**	95.3	94.7	94.9	*94.1*	*93.8*
	Z-scoring	94.7	92.7	93.4	91.0	92.0	91.3	95.4	94.7	**95.0**	93.8	93.2
	Mixture z-scoring	89.1	96.2	92.2	90.3	92.8	91.4	96.0	93.3	94.6	93.5	92.7
**Cross-subject**		**REM**			**Wake**			**NREM**			**Accuracy**	**F1-score**
		**Precision**	**Recall**	**F1-score**	**Precision**	**Recall**	**F1-score**	**Precision**	**Recall**	**F1-score**		
1D-CNN	None	90.9	93.2	**91.8**	89.3	92.7	90.6	95.3	91.5	**93.1**	*92.2*	*91.8*
	Z-scoring	86.7	89.3	86.6	87.4	91.6	88.7	94.1	87.3	89.8	89.1	88.4
	Mixture z-scoring	80.3	89.3	81.1	90.5	91.6	**90.8**	94.2	87.3	89.9	89.1	87.3
AccuSleep	None	84.5	87.4	**85.5**	90.9	88.4	89.3	92.6	92.6	**92.5**	90.8	89.1
	Z-scoring	83.5	84.4	83.3	90.5	88.8	89.4	92.4	92.1	92.1	90.4	88.3
	Mixture z-scoring	75.6	92.4	83.1	90.4	90.6	**90.5**	94.4	90.0	92.1	90.6	88.5
DeepSleepNet	None	92.0	89.9	**89.7**	88.7	93.8	90.9	95.7	91.4	**93.3**	92.2	91.3
	Z-scoring	90.0	87.2	86.8	86.9	92.8	89.0	95.1	88.6	90.9	90.0	88.9
	Mixture z-scoring	81.1	94.9	84.9	89.8	92.9	**91.1**	96.2	87.0	90.6	89.9	88.9

The table reports Precision, Recall, F1-Score, and Accuracy for REM, Wake, and NREM sleep stages. Bold values highlight the best F1-scores for each model across various normalization conditions, whereas underlined and italics values denote the highest overall averaged Accuracy and F1-Score among all models and all conditions.

#### Cross-subject validation.

The 1D-CNN achieved the highest average accuracy and F1-score with raw data, reaching 92.2% and 91.8%, respectively (Table 1, last two columns). Similarly to within-subject validation, no significant differences were found between 1D-CNN and DeepSleepNet. However, both models significantly outperformed AccuSleep in terms of the accuracy and F1-score when raw data were used (F(2, 267) = 17.08, p < 0.001; F(2, 267) = 7.84, p < 0.001, one-way ANOVA, Fig 3C, 3D).

### Class-wise performance

#### Within-subject validation.

**Fig 4:**
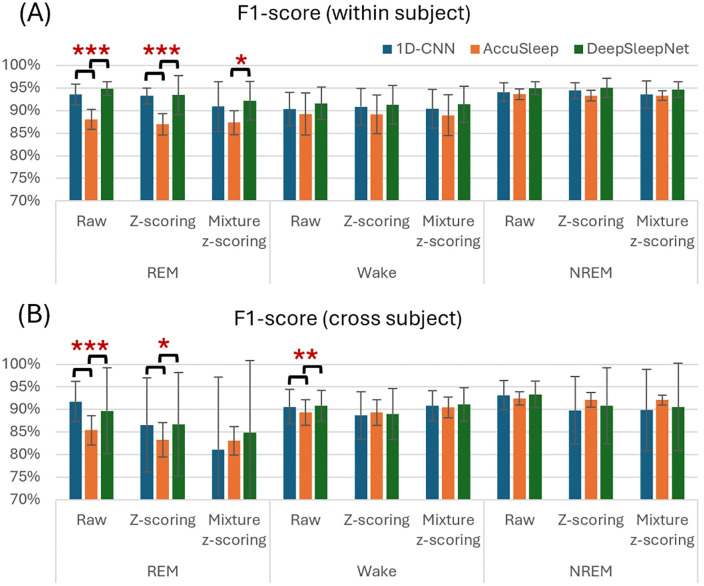
Detailed performance metrics for sleep stage classification across different models and conditions. One-way ANOVA was conducted to compare the performance of the three networks under the same condition (*p < 0.05, **p < 0.01, ***p < 0.001). Each bar represents the mean performance with standard deviation.

No significant differences in F1-scores were found among the models for the Wake and NREM classes. However, for REM classification, the AccuSleep consistently underperformed compared with 1D-CNN and DeepSleepNet when raw or z-scored data were used (F(2, 27) = 31.51, p < 0.001; F(2, 27) = 15.19, p < 0.001, one-way ANOVA, Fig 4A). Furthermore, with mixture z-scoring, the DeepSleepNet demonstrated superior REM classification performance compared with AccuSleep (p = 0.045, post-hoc pairwise comparison, Fig 4A).

#### Cross-subject validation.

No significant differences were observed in NREM classification across models and preprocessing methods (Fig 4B). However, in Wake classification, raw data resulted in significantly higher F1-scores for 1D-CNN and DeepSleepNet compared with AccuSleep (F(2, 267) = 5.34, p = 0.005, one-way ANOVA, Fig 4B). In REM classification, both 1D-CNN and DeepSleepNet consistently outperformed AccuSleep when raw or z-scored data were used (F(2, 267) = 22.94, p < 0.001; F(2, 267) = 4.08, p = 0.018, one-way ANOVA, Fig 4B).

### Impact of normalization methods

#### Within-subject validation.

Normalization had no effect on the accuracy or F1-score, regardless of the model or preprocessing method used (not shown here).

#### Cross-subject validation.

**Fig 5:**
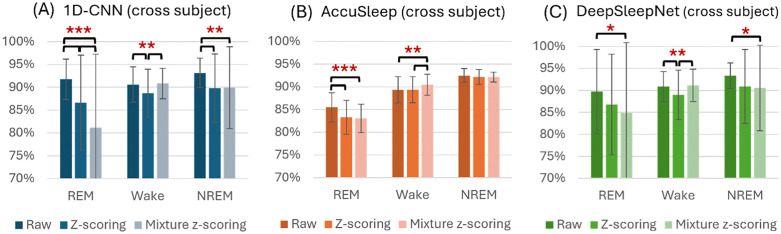
F1-scores of sleep stage classification for each model with cross-subject data across different normalization conditions. One-way ANOVA was conducted to compare the performance of the three networks under the same condition (*p < 0.05, **p < 0.01, ***p < 0.001). Each bar represents the mean performance with standard deviation.

Raw data led to significantly higher F1-scores in REM classification for both 1D-CNN and AccuSleep compared to normalized inputs (1D-CNN: F(2, 267) = 19.66, p < 0.001; AccuSleep: F(2, 267) = 13.84, p < 0.001, one-way ANOVA, Fig 5A, 5B). For Wake classification, AccuSleep with mixture z-scoring outperformed other preprocessing methods (F(2, 267) = 5.13, p = 0.006, one-way ANOVA, Fig 5B). Conversely, z-scoring reduced Wake classification performance in 1D-CNN and DeepSleepNet (F(2, 267) = 6.84, p = 0.001; F(2, 267) = 6.49, p = 0.002, one-way ANOVA, Fig 5A, 5C). For NREM classification, mixture z-scoring resulted in significantly lower F1-scores compared with raw data in 1D-CNN and DeepSleepNet (F(2, 267) = 6.63, p = 0.002; F(2, 267) = 3.55, p = 0.029, one-way ANOVA, Fig 5A, 5C).

### Interpretability of neural network architectures

To interpret the decision-making process of the 2D-CNN model, Gradient-weighted Class Activation Mapping (Grad-CAM) [40] was applied to visualize class-specific evidence in the time-frequency domain. Grad-CAM computes the gradient of the target-class score with respect to the feature maps of a selected convolutional layer and uses the global average of these gradients as weights to produce a coarse localization map highlighting regions that positively contribute to the classification.

Formally, given feature maps *A*^*k*^ of a convolutional layer and the gradient of the score *y*^*c*^ for class *c*, the Grad-CAM heatmap *L*^*c*^_*Grad-CAM*_ is obtained as a weighted combination of feature maps and applying a ReLU activation, retaining only positive contributions.

**Fig 6:**
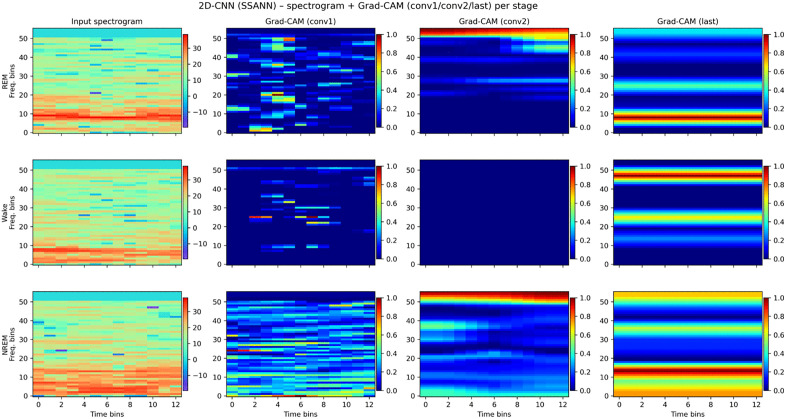
Grad-CAM based saliency maps for the 2D-CNN model. The first column shows spectrograms of representative EEG epochs for each sleep stage. Next two columns display saliency maps of the first and second convolutional layers, and the last column presents the saliency map of the last convolutional layer, illustrating stage-specific frequency preference.

Using Grad‑CAM on the 2D‑CNN, we observed that the later convolutional layers preferentially attended to theta bands for REM, high‑frequency bands for Wake, and low‑frequency bands for NREM (Fig 6), consistent with stage‑specific spectral features.

**Fig 7:**
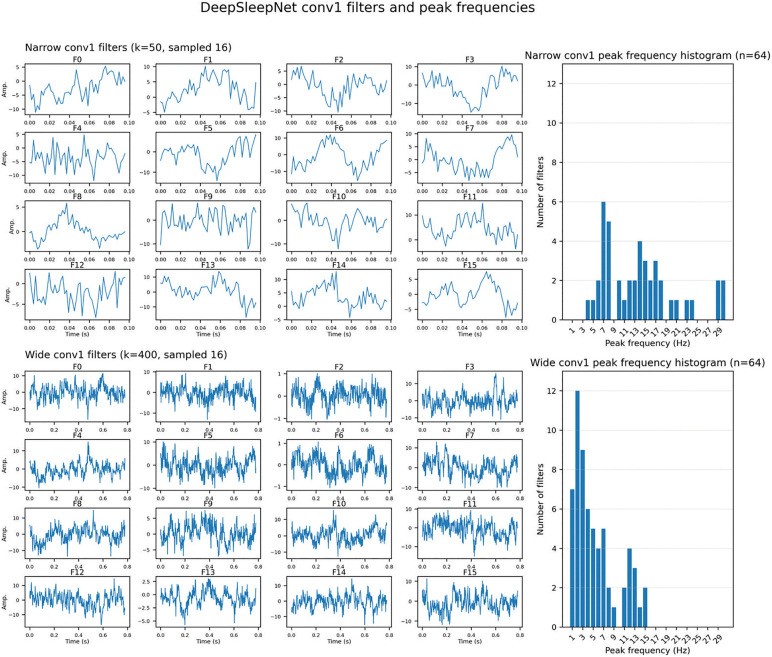
First-layer convolutional filters of DeepSleepNet and their peak frequency distribution. For both narrow and wide convolutional branches, the first 16 filters out of 64 are visualized. The rightmost column shows histogram plots summarize the peak frequencies extracted from all 64 filters, shown separately for narrow and wide convolutional branches. Peak frequency for each filter was defined as the frequency component with the highest amplitude within that filter’s learned weights.

For DeepSleepNet, spectrum analysis of first‑layer convolutional filters revealed peak frequencies clustering in delta–theta–spindle bands for the wide filters, while narrow filters mainly captured temporal patterns (Fig 7). These observations suggest that both architectures exploit stage‑specific spectral cues.

## Discussion

### Model-based comparison for sleep stage classification

This study provides a direct comparison of three representative architectures: 1D-CNN, 2D-CNN (AccuSleep), and CNN with biLSTM (DeepSleepNet) for rodent EEG/EMG sleep staging under identical conditions. Overall, 1D-CNN and DeepSleepNet outperformed AccuSleep across most conditions (Fig 3), suggesting that spectrogram-based models may be disadvantaged in the present setting. AccuSleep’s performance deficit was particularly pronounced in REM, plausibly reflecting class imbalance (REM accounts for approximately 10% of epochs) and the limited time–frequency variability inherent to the dataset. Spectrogram‑based models often benefit from large, diverse datasets that expose richer variability; our dataset, restricted to 10 mice, may not have been sufficiently rich to capitalize on this modeling choice.

Notably, despite biLSTM integration to capture temporal context, DeepSleepNetdid not significantly outperform 1D-CNN on rodent data. Two factors may explain this. First, the limited dataset size likely constrained the LSTM layers’ ability to learn long‑range dependencies. Second, rodents’ polyphasic and fragmented sleep differs from humans’ monophasic, consolidated sleep, potentially making simple CNNs adequate for three‑class staging in rodents [8]. In contrast, human datasets often show a distinct advantage for DeepSleepNet—particularly in N1, a minority stage where temporal context is beneficial [33]. Taken together, these observations indicate that the benefit of temporal modeling is strongly data‑ and species‑dependent.

### Class-wise performance comparison

Class‑wise analyses revealed consistently stable NREM classification across models, whereas REM and Wake were more sensitive to architecture and preprocessing (Fig 4). AccuSleep showed persistently lower REM F1, which likely reflects a combination of class imbalance and spectrogram input limitations. Rem’s characteristic theta activity may be insufficiently expressed in short 2.5-s epochs and small datasets, limiting the spectrogram model’s ability to discriminate. For Wake, AccuSleep underperformed 1D-CNN/DeepSleepNet in cross-subject validation, possibly because the single EMG RMS vector appended to the EEG spectrogram may not capture the full variability of muscle tone and transitions. These findings consolidate the view that dataset scale and diversity critically modulate performance, especially for spectrogram‑based and temporal models. Consistent with this, Yamabe et al. [17] reported markedly improved REM performance for CNN + biLSTM models on large‑scale rodent datasets, whereas smaller datasets showed degraded REM performance—closely mirroring our observations.

### Effects of preprocessing on model performance

Normalization exerted model‑ and class‑specific effects (Fig 5). For 1D-CNN and DeepSleepNet, raw inputs generally yielded the highest performance, while mixture z‑scoring tended to decrease performance in REM and NREM. This suggests that raw EEG/EMG already provide sufficiently informative features, and CNN-based models can effectively learn discriminative patterns without normalization. By contrast, AccuSleep showed a modest Wake improvement under mixture z-scoring, but REM performance deteriorated—indicating that mixture z‑scoring, while designed to address class proportions and subject variability [31], does not guarantee gains across all classes. In sum, normalization is not universally beneficial for rodent sleep staging and may impair performance for CNNs learning from raw signals. Future work should broaden comparisons to include domain adaptation, subject‑aware calibration, and cost‑sensitive losses to determine when normalization helps and when it hinders.

### Insights from literature using different datasets

We summarized the performance of 1D‑CNN and CNN + biLSTM models by incorporating outcomes from related studies alongside our own results to provide broader insight into these architectures (Table 2). Human sleep literature highlight that performance differences are highly dataset‑ and architecture‑dependent. In Sleep‑EDF, DeepSleepNet typically surpasses 1D‑CNN for N1, while 1D‑CNN exceed performance in Wake [32,33], consistent with temporal context aiding minority stages and spectral features sufficing for Wake. Other rodent study underscores the decisive role of data scale: CNN + biLSTM models trained on thousands of mice show improved REM classification [17], whereas smaller datasets yield weaker performance. Our results also show considerable variability in DeepSleepNet’s F1‑score for REM, likely due to the limited number of REM epochs in our small-scale dataset (Fig 5C). These cross‑dataset insights collectively suggest that model choice should be aligned with sleep architecture, class distribution, and dataset scale: simple CNNs can be adequate and robust for rodent three‑class staging, whereas temporal models confer advantages in human datasets or large‑scale rodent cohorts, especially for minority stages.

**Table 2:** F1-scores reported in the literature for 1D-CNN and CNN + biLSTM models applied to rodent and human sleep datasets, alongside results from the present study.

Database		Human data (Sleep-edf)		Rodent data (small-scale)	Rodent data (large-scale)	Rodent data (our data)	
Model		1D-CNN	CNN+biLSTM (DeepSleepNet)	CNN+biLSTM (MC-SleepNet)		1D-CNN	CNN+biLSTM
Wake		98.0	84.7	98.9	97.9	90.6	90.9
NREM	N1	25.0	46.6	96.5	93.1	93.1	93.3
	N2	89.0	85.9				
	N3	87.0	84.8				
REM		81.0	82.4	76.9	84.8	91.8	89.7

### Limitations and future directions

Our evaluation focused on three widely used, reproducible architectures and did not include attention or transformer models [18–20], which remain under‑applied in animal sleep staging. The dataset’s small scale (10 mice) may disadvantage spectrogram‑based models and LSTM components that typically benefit from richer temporal variability. Class imbalance, especially REM (~10%), remained a challenge despite SMOTE, and alternate strategies such as cost‑sensitive learning, focal loss, and curriculum learning may be beneficial. Cross‑species comparisons are limited by differences in class granularity (3 vs. 5) and sleep architecture; multi‑domain representation learning and transfer learning across human/rodent datasets [28,32,33] warrant investigation.

## Conclusions

In rodent EEG/EMG sleep staging, 1D-CNN and CNN + biLSTM models outperformed 2D-CNN under most conditions, with 2D-CNN particularly vulnerable in REM. Although CNN + biLSTM model demonstrates advantages in human datasets, especially for minority stages, its benefit over 1D-CNN was not significant in rodent data, likely reflecting polyphasic sleep and limited dataset size. Raw inputs generally yielded superior performance for CNN/CNN + biLSTM models compared with z‑scored or mixture z‑scored data. Overall, effective sleep staging in rodents favors simpler CNNs and preprocessing choices aligned to data scale, class distribution, and species‑specific sleep architecture. Future work should expand to larger, heterogeneous datasets across species and incorporate attention/transformer architectures to further improve generalizability and interpretability.

## Supporting information

S1 Fig Representative noisy data epochs for each sleep stage. The left column shows three representative raw EEG epochs for each sleep stage, and the right column displays the corresponding EMG signals.
(PNG)
