# Supplementary figures and images for: Neural network architectures and normalization techniques for automated sleep stage classification using rodent EEG and EMG signals
Source: PLoS One. 2026 Apr 23;21(4):e0346294. doi: 10.1371/journal.pone.0346294 (PMC13105341; doi:10.1371/journal.pone.0346294)

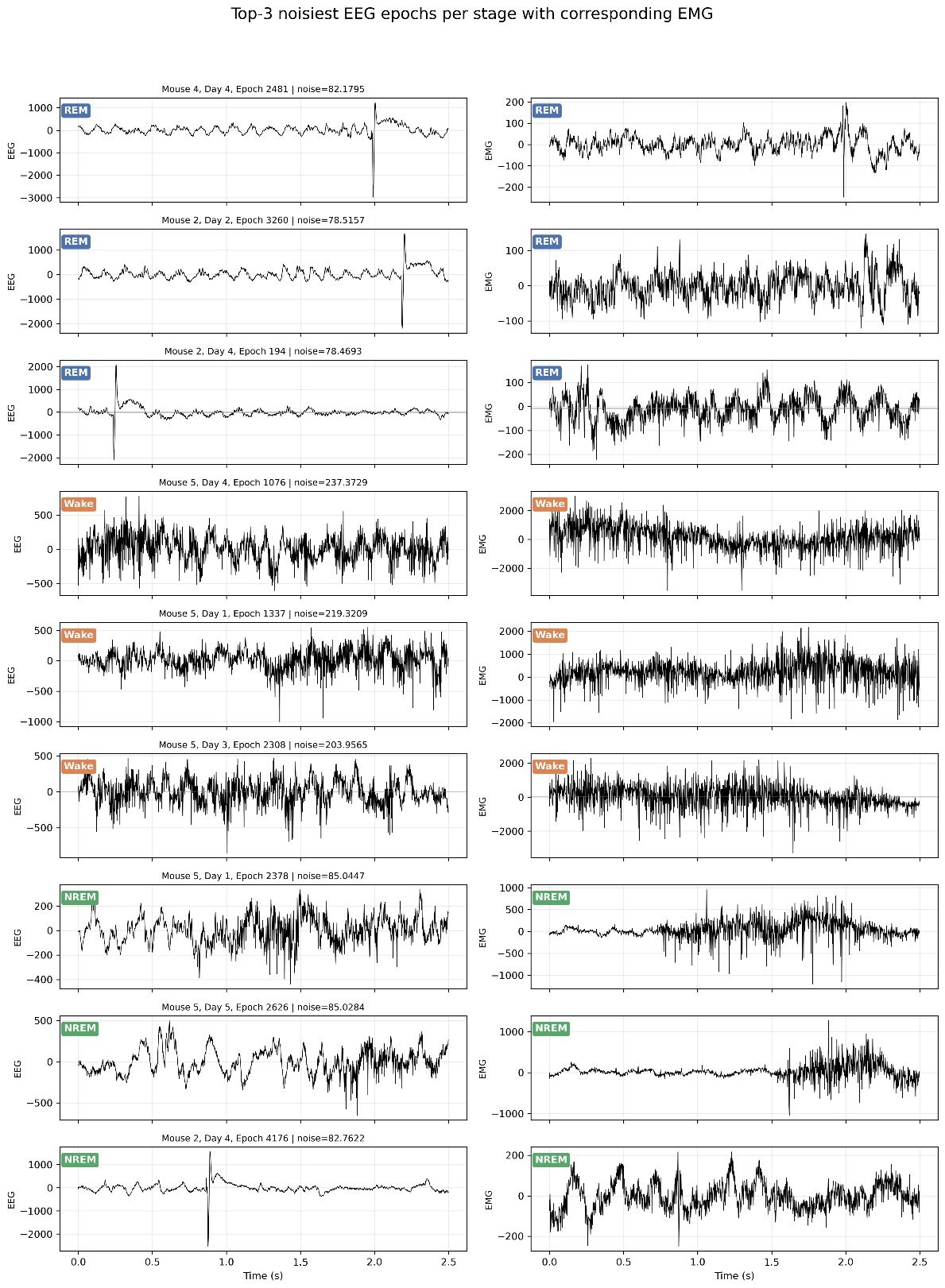

Supplement: S1 Fig — The left column shows three representative raw EEG epochs for each sleep stage, and the right column displays the corresponding EMG signals. (PNG) [file pone.0346294.s001.png]
